# Mortality in people with mental disorders in Poland: A nationwide, register-based cohort study

**DOI:** 10.1192/j.eurpsy.2022.2341

**Published:** 2022-11-18

**Authors:** A. Kiejna, J. Janus, E. Cichoń, S. Paciorek, M. Zięba, T. M. Gondek

**Affiliations:** 1 Department of Psychology, WSB University in Toruń, Toruń, Poland; 2 Department of Psychology, Faculty of Applied Studies, Psychology Research Unit for Public Health, University of Lower Silesia, Wroclaw, Poland; 3 Department of Analyses and Strategy, Ministry of Health, Warsaw, Poland; 4 Institute of Clinical Improvement, Warsaw, Poland; 5 Iter Psychology Practices, Wroclaw, Poland

**Keywords:** Clinical governance, epidemiology, mental disorders, mortality, risk assessment

## Abstract

**Background:**

Mortality among people with mental disorders is higher in comparison with the general population. There is a scarcity of studies on mortality in the abovementioned group of people in Central and Eastern European countries.

**Methods:**

The study aimed to assess all-cause mortality in people with mental disorders in Poland. We conducted a nationwide, register-based cohort study utilizing data from two nationwide registries in Poland: the registry of healthcare services reported to the National Health Fund (2009–2018) and the all-cause death registry from Statistics Poland (2019). We identified individuals who were consulted or hospitalized in public mental healthcare facilities and received at least one diagnosis of mental disorders (International Statistical Classification of Diseases and Health Problems [ICD-10]) from 2009 to 2018. Standardized mortality ratios (SMRs) were compared between people with a history of mental disorder and the general population.

**Results:**

The study comprised 4,038,517 people. The SMR for individuals with any mental disorder compared with the general population was 1.54. SMRs varied across diagnostic groups, with the highest values for substance use disorders (3.04; 95% CI 3.00–3.09), schizophrenia, schizotypal and delusional disorders (2.12; 95% CI 2.06–2.18), and pervasive and specific developmental disorders (1.68; 95% CI 1.08–2.29). When only inpatients were considered, all-cause mortality risk was almost threefold higher than in the general population (SMR 2.90; 95% CI 2.86–2.94).

**Conclusions:**

In Poland, mortality in people with mental disorders is significantly higher than in the general population. The results provide a reference point for future longitudinal studies on mortality in Poland.

## Introduction

Mortality among people with mental disorders is higher than in the general population [1–4]. The highest risks of premature death, from both natural and unnatural causes, are for substance abuse [5, 6] and eating disorders [1].

It is recommended that national policy allows for the collection of data on excess mortality of people with mental disorders [7]. Korkeila et al. list mortality among the key mental health indicators for a health monitoring system in the European Union [8]. Despite that, there is a scarcity of data on mortality in people with mental disorders in Central and Eastern European countries, which makes mortality unavailable for inclusion in the key mental health indicator analyses [9, 10]. Krupchanka et al. reported significantly higher mortality among people with mental disorders in comparison with the general population in the Czech Republic [11]. In the Czech population, all analyzed ICD-10 diagnostic groups for mental disorders were characterized by markedly elevated standardized mortality ratios (SMRs), with the highest SMR for substance use disorders [11]. Bitter et al. showed that people with schizophrenia have a significantly higher risk of all-cause mortality than the general population in Hungary [12]. There is a lack of studies on mortality in people with mental disorders in Poland.

In psychiatric care in Poland, there is currently a shift from institutional to community-based care. The National Mental Health Protection Programme for the years 2017–2022 has been adopted in Poland and its objectives include the promotion and implementation of the community-based model of psychiatric healthcare [13]. One of the tasks of the program is to improve the effectiveness of the system indices, which include mortality. The availability of health data registries allows for large-group comparisons, and a registry-based study in the Nordic countries showed that during the period of deinstitutionalization, the life expectancy gap between people with mental illness and the general population had been reduced [14]. It may have partially been explained by the reduction of the suicide rates [15]. This study is the first to use Poland’s National Health Fund data registry to measure mortality rates of people diagnosed with mental disorders. The available registry allowed the analysis of data for both outpatients (consulted in either mental health clinics or mental health day treatment facilities) and inpatients of public mental healthcare facilities in Poland.

## Methods

### Study design

We conducted a cohort study based on the group-level linkage of registered nationwide data collected for one decade (from January 1, 2009 to December 31, 2018) in Poland and deaths in 2019.

We used two nationwide registries: (a) healthcare services reported to the National Health Fund (NHF) and (b) death registry from Statistics Poland (SP).

The NHF’s registers are part of data of the Central Insurance List collected for monitoring and managing service provision in Poland. The database contains patients’ national identification numbers (PESEL numbers) and information about services provided to patients throughout Poland. We considered the services provided as a result of diagnoses in the group “Mental and behavioral disorders” (according to the International Statistical Classification of Diseases and Health Problems [ICD-10]). These data are sent directly to the NHF as a standard registration form by healthcare professionals. The diagnosis is made by a psychiatrist after an outpatient consultation or during hospitalization.

Patients were classified according to the most intensive form of lifetime treatment present in the registry: (a) Inpatients—hospitalized at least once in an inpatient unit; (b) Outpatients-day care center—hospitalized at least once in a day care center but never hospitalized in an inpatient unit; and (c) Outpatients-clinic/consultations—received outpatient psychiatric care but never hospitalized in either inpatient or day care center.

The second database including PESEL numbers and dates of death is based on death certificates mandatorily filled out by physicians and submitted to the registry office for all deaths in Poland. We took into account all-cause mortality in 2019 (all deaths occurring between January 1, 2019 and December 31, 2019) for individuals with and without a history of using healthcare services due to psychiatric diagnoses.

This study was approved by the Ethical Committee of the University of Lower Silesia (opinion no. 2/2021). The study procedures adhered to recognized research standards as outlined by the Declaration of Helsinki.

### Data preparation

#### Data linkage

We used the database of all-cause healthcare services reported to the NHF to identify all individuals receiving healthcare services due to diagnosis of mental and behavioral disorders between January 1, 2009 and December 31, 2018. We linked these data with the all-cause mortality database from SP. The record linkage was carried out with the use of the encoded PESEL number.

#### Exclusion/inclusion criteria and data cleaning

The presented analysis concerns services due to diagnosis of mental and behavioral disorders provided to persons who were 15–85 years old in 2019. The diagnoses of mental disorders have been divided into 10 groups according to ICD-10 codes:

1. F10–F19: Mental and behavior disorders due to psychoactive substance use
2. F20–F29: Schizophrenia, schizotypal, delusional, and other non-mood psychotic disorders
3. F30–F39: Mood (affective) disorders
4. F40–F48: Anxiety, dissociative, stress-related, somatoform, and other nonpsychotic mental disorders
5. F50–F59: Behavior syndromes associated with physiological disturbances and physical factors
6. F60–F69: Disorders of adult personality and behavior
7. F80–F89: Pervasive and specific developmental disorders
8. F90–F98: Behavior and emotional disorders with onset usually occurring in childhood and adolescence
9. F99–F99: Unspecified mental disorder
10. Individuals with a history of multiple diagnoses, that is, patients who received more than one diagnosis during 2009–2018 (see Supplementary Table 1a for detailed comorbidity distribution in this group and Supplementary Table 1b for the distribution of individual diagnoses in this group).

We excluded the missing data associated with persons who were not identified according to their PESEL number at the time of the service provision (no identity document at the time of the service provision or no PESEL number assigned) due to no possibility to follow-up on their later status. The data of individuals who died before January 1, 2019 were also rejected. Other exclusion criteria were intellectual disabilities (ICD-10: F70–F79) or organic, including symptomatic and mental disorders (F00–F09) due to premature death inherent to these disorders. The exclusion of F00–F09 and F70–F79 is common in mortality studies [11, 14, 16–19]. The selection procedure was presented in the flow diagram (Supplementary Figure 1).

### Data analysis

All-cause mortality in 2019 (January 1, 2019–December 31, 2019) was analyzed for individuals with and without a history of receiving healthcare due to psychiatric diagnoses (Supplementary Figure 1). The SMR with 95% CI was calculated based on age- and sex-specific death rates for each group of mental disorders and for individuals with a history of multiple diagnoses. SMR is the ratio of the observed number of deaths (or cases of any incidents) in the cohort to the number of deaths (or incident cases) that would be expected in the general population [20]. The percentage distribution of deaths was plotted by age for each diagnostic category, for the whole group, separately for men and women, and for the general population. χ² tests were used for comparisons of age distributions in the general population. Finally, the SMRs with 95% CI for each diagnostic category and for the whole group according to the type of healthcare (inpatient, outpatient-clinic, and outpatient-day care center) were estimated. All statistical analyses were conducted in the R software (version 3.6.1).

## Results

The final study population comprised 4,038,517 individuals (Supplementary Figure 1). Sociodemographic characteristics of the study population are summarized in Supplementary Table 2. The mean age was 40.82 years (SD = 15.99). The proportion of men to women was similar (χ^2^(1) = 31,506.67; *p* = 0.064). F40–F48 (34.66%) and F10–F19 (17.57%) were the two major subgroups, followed by F30–F39 (10.59%) and F20–F29 (4.54%). Up to 25.44% (*n* = 1,027,347) of people had a history of multiple diagnoses of mental and behavioral disorders.

**Figure 1.**
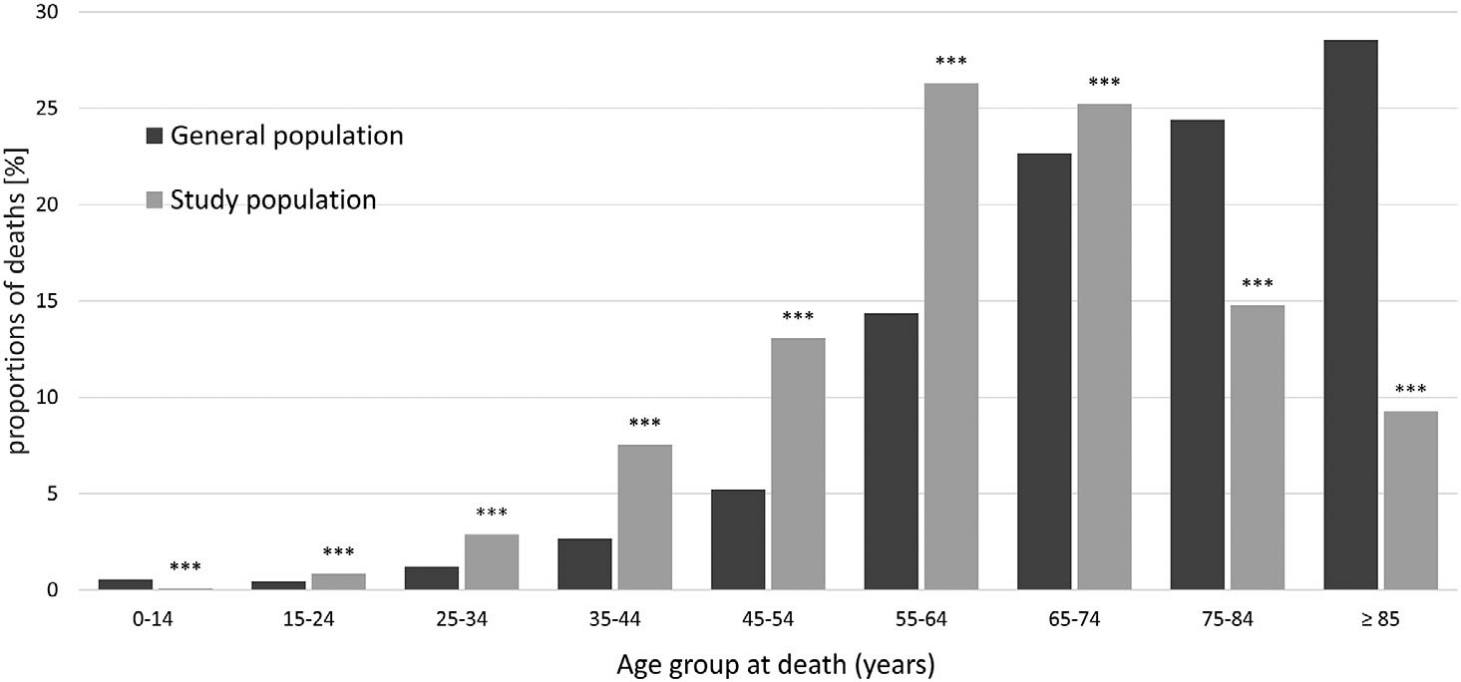
Age at death in the study population versus the general population in 2019. The proportions of deaths in 2019 in each age group among people with a history of healthcare use due to psychiatric diagnoses (according to ICD-10) between January 1, 2009 and December 31, 2018, were contrasted with the proportions of deaths in each age category in the general population. ***χ² goodness of fit test showed significant differences between the study population and the general population (*p* < 0.001).

A total of 50,654 individuals (61.98% males) within the study population died between January 1, 2019 and December 31, 2019 (Supplementary Table 2). In 2019, individuals who had received services for any mental disorder were significantly younger at death than those in the general population (*p* < 0.001; Figure 1). This difference was also observed when men and women were compared separately (*p <* 0.001; Supplementary Figure 2). Generally, the age group with the highest proportion of deaths among those with a history of mental illness was 55–64 years, whereas in the general population, the highest proportion of deaths was observed for people 20 years older (>84 years) (Figure 1). When sex differences were considered, the highest proportion of deaths among males in the study population was also for 55–64 years, whereas it was >85 years in the general population. However, the peak of deaths among females in the study population was observed for 65–74 years, whereas in the general population it was observed for >85 years (Supplementary Figure 2).

There was a significant difference between the study population and the general population (*p <* 0.001) for each age group (Supplementary Table 3). These differences were also shown for each age/sex category (*p* < 0.001) (Supplementary Table 3).

**Figure 2.**
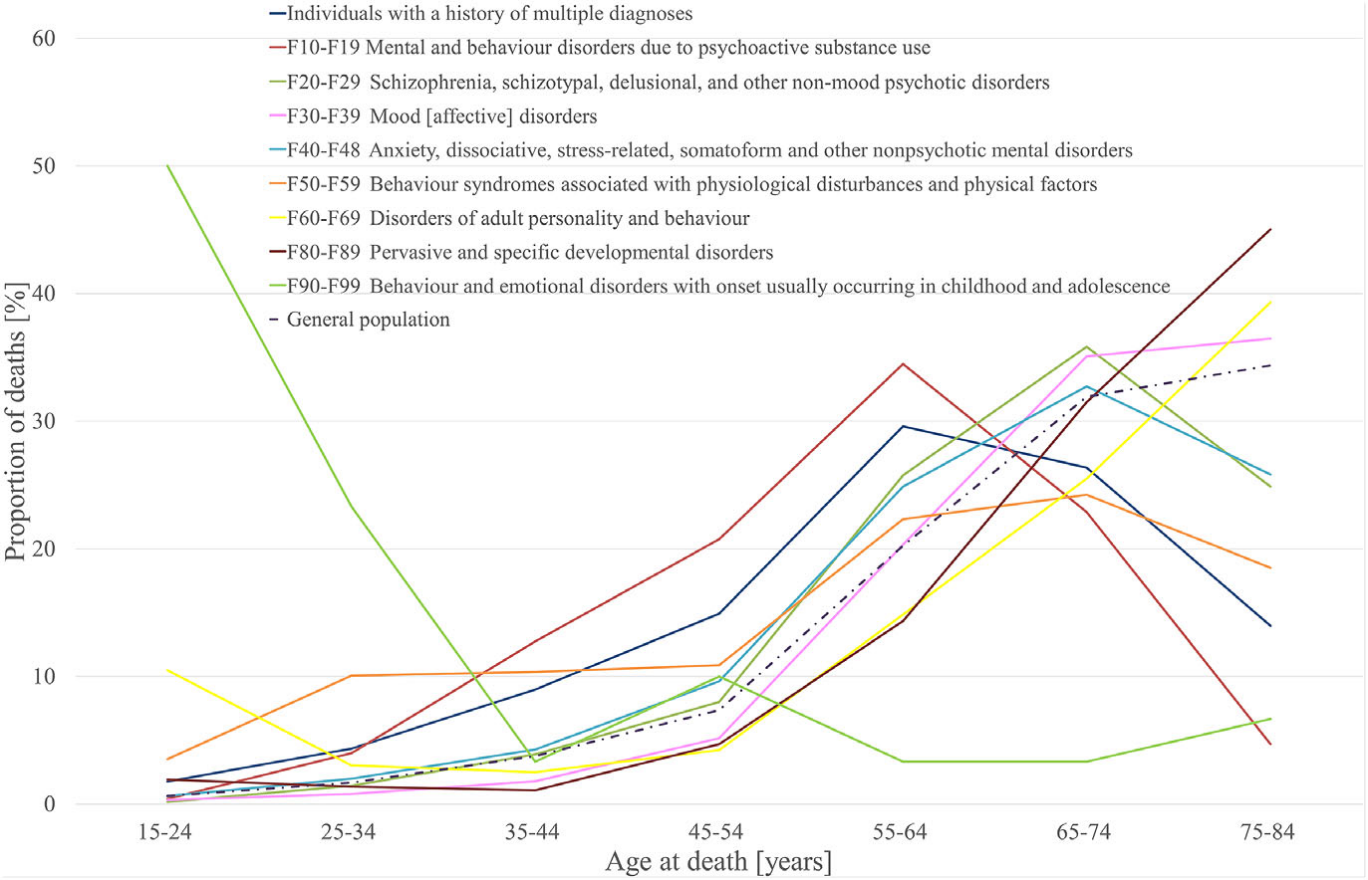
Age at death in the general population in 2019 and among each diagnostic category of the study population.

Standardized annual mortality ratios with 95% CIs and the proportion of deaths for individuals with any category of age and sex according to the settings of treatment compared with the general population in 2019 are presented in Table 2 and in Supplementary Table 4. Distributions of age at death for each diagnostic group are presented in Figure 2, and separately for men and women in Supplementary Table 4.

In general, peaks in mortality for most of the disorders have occurred at an earlier age than in the general population. However, the highest mortality peaks for behavior syndromes associated with physiological disturbances and physical factors (F50–F59), mood (affective) disorders (F30–F39), and behavior and emotional disorders with onset usually occurring in childhood and adolescence (F90–F99) were attained at the age of 75–84 years, which was also true for the general population.

The χ² of proportions rejected the null hypothesis of no difference in the distribution of data in age categories when comparing each diagnostic category separately with the general population (*p* < 0.001).

The distribution of deaths according to treatment settings indicates in general that beneficiaries of psychiatric and addiction care or mental health centers in each treatment category are more likely to die than members of the general population (Table 1). Among outpatients-clinic, outpatients-day care center, and inpatients, the observed deaths were 112, 134, and 290% of the expected mortality, respectively. Interestingly, both females and males at the age of 15–34 and 65–84 in the day care center were not significantly more likely to die than expected based on the mortality rates of the general population. The same non-significant SMR was observed for 65- to 74-year-old females in the outpatients—clinic setting. The SMR was significantly lower for 75- to 84-year-old outpatients—clinic (regardless of gender) and significantly higher for the rest of the sex/age groups in all treatment settings. The highest SMR was observed for 35–44 years old female inpatients. The SMR of 9.70 (95% CI 8.79–10.60) in this group indicated that more than nine times as many deaths were observed than expected based on the general population. For younger female inpatients (25–34 years), the SMR of 8.89 also indicated a very high mortality rate in comparison to the general population. The highest SMR values in males were also observed for this age/treatment setting groups (25–34 years: 6.39 [95% CI 5.97–6.81]; 35–44 years: 5.91 [95% CI 5.66–6.15]).

**Table 1.** Distribution of mortality in 2019 among the study population according to age, sex, and settings of treatment.

	Outpatients − clinic/consultations		Outpatients − day care center		Inpatients	
Female	Deaths *N* (%)^a^	SMR (95% CI)	Deaths *N* (%)^a^	SMR (95% CI)	Deaths *N* (%)^a^	SMR (95% CI)
15–24	59 (0.22%)	1.69 (1.26–2.12)	5 (0.48%)	5.04 (0.62–9.45)	54 (0.24%)	6.74 (4.95–8.54)
25–34	135 (0.50%)	1.42 (1.18–1.66)	6 (0.58%)	1.54 (0.31–2.77)	134 (0.60%)	8.89 (7.39–10.40)
35–44	431 (1.58%)	1.37 (1.24–1.50)	32 (3.08%)	2.29 (1.50–3.08)	442 (1.98%)	9.70 (8.79–10.60)
45–54	1055 (3.87%)	1.30 (1.22–1.38)	64 (6.16%)	1.86 (1.41–2.32)	756 (3.38%)	5.46 (5.07–5.84)
55–64	2948 (10.82%)	1.12 (1.08–1.16)	120 (11.55%)	1.24 (1.02–1.46)	1701 (7.61%)	3.19 (3.04–3.35)
65–74	4141 (15.20%)	1.02 (0.99–1.05)	82 (7.89%)	1.11 (0.87–1.34)	1753 (7.84%)	2.58 (2.46–2.70)
75–84	3999 (14.68%)	0.92 (0.90–0.95)	46 (4.43%)	0.83 (0.59–1.07)	1297 (5.80%)	1.85 (1.75–1.95)
Male						
15–24	182 (0.67%)	1.45 (1.24–1.66)	3 (0.29%)	0.64 (−0.08–1.36)	167 (0.75%)	5.84 (4.95–6.72)
25–34	430 (1.58%)	1.57 (1.42–1.72)	21 (2.02%)	1.62 (0.93–2.32)	888 (3.97%)	6.39 (5.97–6.81)
35–44	1029 (3.78%)	1.59 (1.49–1.69)	65 (6.26%)	1.82 (1.38–2.26)	2226 (9.95%)	5.91 (5.66–6.15)
45–54	1964 (7.21%)	1.44 (1.38–1.51)	129 (12.42%)	1.47 (1.21–1.72)	3344 (14.95%)	3.99 (3.86–4.12)
55–64	4499 (16.51%)	1.26 (1.22–1.30)	263 (25.31%)	1.48 (1.30–1.66)	5169 (23.11%)	2.59 (2.52–2.66)
65–74	4421 (16.22%)	1.09(1.06–1.12)	163 (15.69%)	1.15 (0.97–1.32)	3523 (15.75%)	2.13 (2.06–2.20)
75–84	1957 (7.18%)	0.93 (0.89–0.97)	40 (3.85%)	1.16 (0.80–1.52)	911 (4.07%)	1.64 (1.54–1.75)
SMR (95% CI)^c^	1.12 (1.10–1.13)		1.34 (1.26–1.42)		2.90 (2.86–2.94)	
Total deaths (%)^b^	27,250 (53.80%)		1039 (2.05%)		22,365 (44.15%)	
	50,654 (100%)					

a Percentages of deaths for each treatment settings/age/sex group within all deaths in the treatment settings category.

b Percentages of all deaths for each treatment settings category within all deaths in the study population.

c SMR for all categories of treatment settings.

The overall SMR for individuals with any mental disorder compared with the general population was estimated at 1.54 (95% CI 1.53–1.55) and varied across diagnostic groups (Table 2). The highest ratios were for mental and behavioral disorders due to psychoactive substance use (F10–F19; SMR 3.04; 95% CI 3.00–3.09); schizophrenia, schizotypal, delusional, and other non-mood psychotic disorders (F20–F29; 2.12; 95% CI 2.06–2.18); and pervasive and specific developmental disorders (F80–F89; 1.68; 95% CI 1.08–2.29); followed by behavior and emotional disorders with onset usually occurring in childhood and adolescence (F90–F99; 1.39; 95% CI 1.30–1.50) and disorders of adult personality and behavior (F60–F69; 1.24; 95% CI 1.11–1.37). SMR values for mood (affective) disorders were slightly higher than for the general population (1.08; 95% CI 1.05–1.11). Anxiety, dissociative, stress-related, somatoform, and other nonpsychotic mental disorders, as well as behavior syndromes associated with physiological disturbances and physical factors, had a lower SMR than the general population (0.78, 95% CI 0.77–0.80; 0.84, 95% CI 0.75–0.93, respectively). However, if types of treatment were considered, in contrast to outpatients, SMR for inpatients was significantly higher than that in the general population (Supplementary Table 4). Those with a history of multiple diagnoses had an estimated SMR of 1.45 (95% CI 1.43–1.48; Table 2).

**Table 2.** Standardized annual mortality ratios of the study population in comparison with the general population in 2019.

		SMR	95% CI
General population		1 (ref.)	–
Any diagnoses		1.54	1.53–1.55
F10–F19	Mental and behavior disorders due to psychoactive substance use	3.04	3.00–3.09
F20–F29	Schizophrenia, schizotypal, delusional, and other non-mood psychotic disorders	2.12	2.06–2.18
F30–F39	Mood (affective) disorders	1.08	1.05–1.10
F40–F48	Anxiety, dissociative, stress-related, somatoform and other nonpsychotic mental disorders	0.78	0.77–0.80
F50–F59	Behavior syndromes associated with physiological disturbances and physical factors	0.84	0.75–0.92
F60–F69	Disorders of adult personality and behavior	1.24	1.11–1.36
F80–F89	Pervasive and specific developmental disorders	1.68	1.08–2.29
F90–F99	Behavior and emotional disorders with onset usually occurring in childhood and adolescence	1.39	1.29–1.49
	Individuals with a history of multiple diagnoses	1.45	1.43–1.48

## Discussion

The results of the study indicate that people with a diagnosis of a mental disorder who were diagnosed with any mental disorder had a higher SMR in 2019 in comparison with the general population in Poland. Significantly increased SMRs were found for all analyzed ICD-10 diagnostic groups except: F40–F48 and F50–F59.

Direct comparisons of results between different studies are difficult due to differences of methodology: differences in the population studied, discrepancies in the data included in the registers used and in the quality of the data collected in the registers. A summary of the results of other studies was presented in Supplementary Table 5.

A recent study from the Czech Republic with an analogous methodology showed significantly higher SMRs for inpatients from all diagnostic groups in comparison with the general population [11]. SMR for any diagnosis was 2.2 (95% CI 2.2–2.3), whereas in our study SMR for inpatients was significantly higher, 2.9 (95% CI 2.86–2.94). SMR for any diagnosis in the total study group in Poland, including both outpatients and inpatients, was markedly lower at 1.54 (95% CI 1.53–1.55). The higher overall SMRs were also observed in the Italian cohort studies including inpatients and outpatients during a 10-year observation period: 1.99 and 1.8 [21, 22]. In the Danish cohort study including all persons with mental disorders younger than 95 years, mortality due to all causes was more than two times higher among people diagnosed with a mental disorder compared with those without any diagnosis (adjusted MRR 2.53, 95% CI 2.52–2.54), whereas study carried out in the Netherlands, comparing public mental health care clients aged 15 or more to matched controls, indicated hazard ratio (HR) value of 2.99 (95% CI 2.63–3.41) [6, 23]. A meta-analysis by Walker et al. showed that the relative risk for all-cause mortality in people with mental disorders was 2.22 (95% CI 2.12–2.33) [4]. Thus, the overall SMR for the total group of patients with mental disorders in Poland was lower in comparison with other European countries.

Interestingly, outpatients consulted in either day care centers or outpatient clinics with F40–F48 diagnosis, outpatients consulted in outpatient clinics with F50–F59 diagnosis, and outpatients consulted in day care centers with F30–F39 diagnosis had lower SMR than the general population. However, considering inpatients only, SMR was significantly higher in all diagnostic groups in comparison with the general population. Higher mortality rates among inpatients compared with the general population are expected, because inpatients tend to have more comorbid somatic illness than the general population [24, 25]. Premature death among patients with severe psychiatric disorders may be caused by side effects of medication, unhealthy lifestyles, and possible genetic vulnerability [26]. Psychiatric patients are less likely to seek medical care, have limited access to care or receive lower quality physical health care, thus medical problems often remain undetected and undertreated [26]. Some researchers underline over-representativeness of hospitalized psychiatric patients who often have more severe illness and that mortality in persons with mental disorders seems to be overestimated by inpatient psychiatric diagnoses [27]. Indeed, most studies included inpatients only [4]. Moreover, the studies including inpatients and outpatients mostly did not estimate all-cause SMRs separately for these groups in reference to the general population [4, 27–30]. In fact, we found only a few studies that provide all-cause SMRs separately for outpatients and inpatients [31–34].

For example, in the Czech study including only inpatients in the analysis, SMR for anxiety, dissociative, stress-related, somatoform, and other nonpsychotic mental disorders (F40–F48) was 1.8 (1.6–1.9), whereas in our study SMR for this group was 1.44 (95% CI 1.34–1.53) [11]. Our analysis showed that inpatients and outpatients diagnosed with F40–F48 had in total a lower SMR than the general population, at 0.78 (95% CI 0.77–0.80). A closer analysis indicated that the outpatients with a diagnosis of F40–F48 had lower SMR than the general population in all age groups, apart from females aged 15–24. However, for inpatients with a diagnosis of F40–F48, overall SMR was significantly higher than in the general population. In our study, lower SMRs were also observed among outpatients with F30–F39 and F50–F59 diagnoses.

A fewer than expected number of deaths were also observed in the U.S. study on 500 outpatients followed-up for a mean time of 7 years: Martin et al. reported lower SMRs for both primary unipolar (0.84) and bipolar affective disorder (0.62), as well as for persons with a diagnosis of hysteria (0.77), in comparison with the general population [31]. Patients with primary anxiety neurosis did not present excess mortality in reference to the general population (SMR = 1.03) [31]. Prior reported significantly raised all-cause relative risk (RR) for depression but not for anxiety states and personality disorders among outpatients [33]. Park showed that for patients in Korea who received outpatient care, the mortality risks were 1.6- and 1.5-fold higher than for men and women in the general population, respectively [34]. In that study, all psychiatric diagnoses except neurotic disorders were associated with significant excess mortality both for outpatients and inpatients [34]. However, for outpatients with neurotic disorders SMR was significantly lower than in the general population (0.86; 95% CI: 0.78–0.95). Considering these results and our data, it seems that outpatients with anxiety disorders may have lower mortality risk than the general population.

The tendency to worry characteristic for patients with anxiety and mood disorders may lead to self-care behaviors and security seeking [35]. It may be hypothesized that outpatients diagnosed with F40–F48, due to the nature of anxiety disorders, tend to seek contact with healthcare professionals more frequently and thus may be diagnosed with comorbid disorders and receive treatment earlier. Similarly, people with F50–F59 diagnosis in our study were characterized by a significantly lower SMR than the general population, at 0.84 (95% CI 0.75–0.92). However, a markedly higher SMR was observed in inpatients with an F50–F59 diagnosis, reaching 3.60 (2.24–4.96), which was the second highest SMR among all diagnostic groups when only inpatients were considered. The presented differences between outpatients and inpatients may result from a relatively smaller population diagnosed with F50–F59, compared with the majority of other diagnostic groups, particularly regarding inpatients with these diagnoses.

In our study, the highest SMR was observed for inpatients with F10–F19 diagnosis and it reached 3.77 (95% CI 3.70–3.84). In other studies, individuals with substance use disorders were also characterized by the highest SMR among all diagnostic groups [6, 11, 18, 22]. In our study, the total population with substance use disorders had an SMR of 3.04 (3.00–3.09), which was lower than in a prospective longitudinal study of patients followed for 19 years in Norway, for which an SMR of 3.8 (95% CI 3.2–4.6) was observed [36].

The Czech study revealed that SMR for F20–F29 diagnosis was 2.3 (95% CI 2.1–2.5), whereas, in our study, SMR for inpatients was 2.6 (95% CI 2.50–2.71) [11]. However, considering both in- and outpatients in our study, people with the diagnosis of F20–F29 were characterized by an SMR of 2.12 (95% CI 2.06–2.18). In contrast, in a Hungarian prospective matched-cohort study, people with a diagnosis of schizophrenia between 2005 and 2013 had a higher all-cause mortality rate (RR = 2.4) [12]. Similar findings were reported among Danish in- and outpatients, where mortality rate ratio (MRR) for F20–F29 was 2.57 (2.54–2.60) [6]. A recent meta-analysis indicated a higher SMR, at 3.08 (95% CI 2.88–3.31), for schizophrenia and other psychotic disorders [37]. Thus, in Poland mortality rate for people with F20–F29 diagnosis was relatively low in comparison to other countries. Similar pattern of the results was found in people with mood (affective) disorders. The SMR for F30–F39 diagnosis among inpatients in Czech Republic was 1.6 (1.5–1.7) and was close to our results for inpatients with these disorders (1.69; 95% CI 1.59–1.78) [11]. However, when all patients diagnosed with mood disorders were considered, SMR in our study was 1.08 (95% CI 1.05–1.10). A prospective study of outpatients with affective disorders during a 5-year follow-up in Germany showed a markedly higher SMR of 2.9 (95% CI 1.8–4.0) overall in people with affective disorders [38]. A meta-analysis by Walker et al. estimated all-cause mortality rate in people with F30–F39 diagnoses of 2.08 (1.89–2.30) [4]. A lower mortality rate, but still higher in comparison with our study, was observed in a Danish study among in- and outpatients where MRR for mood (affective) diagnosis was 1.92 (95% CI 1.91–1.94) [6].

In the Czech study [11], SMR for disorders of adult personality and behavior (F60–F69) was 2.3 (95% CI 2.0–2.6), while in the Polish study SMR for inpatients reached 2.55 (95% CI 2.00–3.09) [11]. When all patients were considered, SMR was 1.24 (95% CI 1.11–1.36). In other studies, SMRs for personality disorders were significantly higher. Kuo et al. reported that SMR for all-cause mortality in persons with personality disorder, between 1985 and 2008, was markedly higher, 4.46 (95% CI 1.94–6.98), whereas in a study by Fok et al. SMR in persons with personality disorders within secondary mental health care was 4.2 (95% CI 3.03–5.64) [39, 40].

Individuals with a history of multiple diagnoses in the Czech Republic had SMR at 2.9 (95% CI 2.7–3.2), whereas in Poland SMR in this group was 2.55 (95% CI 2.49–2.62) and for all patients it was 1.45 (1.43–1.48) [11].

One of the limitations of the study was no technical or legal possibility to link the national health data registry run by the NHF with the registry of causes of death, run by SP. Therefore, calculation of cause-specific mortality rates was not possible. In addition, due to the low number of deaths in the 0–14 age group during the adopted observation period, the methodology adopted would not have been suitable to assess mortality in this group. The reported lower death rates in some categories in Poland as compared to other countries may also be related to the exclusion of 17,922 patients in Poland due to errors or missing data and therefore no possibility of linking their data between the healthcare services registry and death registry.

The study sample only comprised patients either consulted or hospitalized in public mental healthcare facilities, there was no access to data of patients consulted or hospitalized only in private mental healthcare facilities or those consulted in primary care or in public specialist clinics but never consulted or hospitalized in mental healthcare facilities. Due to the costs associated with the use of private care, people with greater social disabilities, and therefore those with diagnoses of severe mental disorders, will generally have less access to such care and are more likely to use public care. A register collecting data only from public care facilities may therefore have contained slightly fewer patients with diagnoses of less severe disorders.

On the other hand, according to Ûstùn and Sartorius [41], the most common mental disorders in primary care are: depression (10.4%), generalized anxiety disorder (7.9%), and neurasthenia (5.4%). According to Roca et al. [42] the most prevalent psychiatric disorders in primary care were: affective (35.8%), anxiety (25.6%), and somatoform (28.8%) disorders. In Poland, access to a psychiatrist is not systemically restricted: a referral from a general practitioner is not required. However, persons presenting themselves to a GP whose mental state requires it are referred to an outpatient mental health clinic. Some of the patients, mainly with depressive disorders (of lesser severity), anxiety or somatoform disorders, whose condition did not require referral to a psychiatrist and who used only primary care, may not have been included in the study.

A reform of mental health care is currently taking place in Poland, involving the integration of mental health services with primary health care, the development of community-based psychiatric services and a reduction in the role of psychiatric hospitals. The overall aim is to shift the healthcare model from institution-based care toward community-based care. A pilot program of Mental Health Centers is being developed, and there are now 75 such centers across Poland, providing psychiatric care for adults living in a defined territory. The results of a recent study analyzing health care effectiveness indicators indicate that the first phase of the pilot of the Mental Health Centers, involving 27 centers, is delivering the intended outcomes, based on the aims set in The National Mental Health Protection Programme for the years 2017–2022 [43]. The program is expected to be continued in the following years.

Our study presents all-cause mortality risk for individuals with mental disorders before the COVID-19 outbreak. As the first such study in Poland, it paves the way for establishing the continuous monitoring of one of the key mental health indicators, mortality, in people with mental disorders, as outlined in the National Mental Health Protection Programme for the years 2017–2022 [13]. The obtained results will serve as a reference point for future longitudinal mortality studies in Poland.

## Data Availability

Data were generated at a central, large-scale facility, available upon request. Raw data were generated at Department of Analyses and Strategy, Ministry of Health, Warsaw, Poland. Derived data supporting the findings of this study are available from the corresponding author SP on request.

## Supplementary material

For supplementary material accompanying this paper visit https://doi.org/10.1192/j.eurpsy.2022.2341. click here to view supplementary material
