# Supplementary material for: Mortality in people with mental disorders in Poland: A nationwide, register-based cohort study
Source: Eur Psychiatry. 2022 Nov 18;66(1):e2. doi: 10.1192/j.eurpsy.2022.2341 (PMC9879895; doi:10.1192/j.eurpsy.2022.2341)
Supplement: Supplementary file 1 [file S0924933822023410sup001.zip › S0924933822023410sup004.docx]

**Supplementary Table 1a**

*The distribution of comorbidities in the group of patients with a history of multiple diagnoses*

| Individuals with a history of multiple diagnoses (during 2009-2018) | | | | | |
| --- | --- | --- | --- | --- | --- |
| I | II | III | n | % | Cumulative % |
| F30-F39 | F40-48 |  | 325262 | 31,66% | 31,66% |
| F40-48 | F10-F19 |  | 133185 | 12,96% | 44,62% |
| F40-48 | F90-F98 |  | 59425 | 5,78% | 50,41% |
| F40-48 | F60-69 |  | 49961 | 4,86% | 55,27% |
| F30-F39 | F40-48 | F10-F19 | 37679 | 3,67% | 58,94% |
| F20-F29 | F30-39 |  | 35408 | 3,45% | 62,39% |
| F30-39 | F10-F19 |  | 34693 | 3,38% | 65,76% |
| F20-F29 | F40-48 |  | 28575 | 2,78% | 68,54% |
| F30-39 | F40-48 | F60-69 | 27528 | 2,68% | 71,22% |
| F60-69 | F10-F19 |  | 21160 | 2,06% | 73,28% |
| F20-F29 | F10-F19 |  | 19550 | 1,90% | 75,19% |
| F20-F29 | F30-39 | F40-48 | 18294 | 1,78% | 76,97% |
| F30-39 | F40-48 | F90-F98 | 16194 | 1,58% | 78,54% |
| F40-48 | F50-59 |  | 15780 | 1,54% | 80,08% |
| F30-39 | F90-F98 |  | 15025 | 1,46% | 81,54% |
| F80-89 | F90-F98 |  | 14890 | 1,45% | 82,99% |
| F40-48 | F60-69 | F10-F19 | 12911 | 1,26% | 84,25% |
| F10-F19 | F90-F98 |  | 12387 | 1,21% | 85,45% |
| F30-39 | F60-69 |  | 11835 | 1,15% | 86,61% |
| Comorbidity with <1% frequency | | | 137605 | 13,39% | 100,00% |
