# Supplementary material for: Mortality in people with mental disorders in Poland: A nationwide, register-based cohort study
Source: Eur Psychiatry. 2022 Nov 18;66(1):e2. doi: 10.1192/j.eurpsy.2022.2341 (PMC9879895; doi:10.1192/j.eurpsy.2022.2341)
Supplement: Supplementary file 1 [file S0924933822023410sup001.zip › S0924933822023410sup005.docx]

**Supplementary Table 1b**

*The distribution of individual diagnoses in the group of patients with a history of multiple diagnoses*

| Individuals with a history of multiple diagnoses (during 2009-2018) | | |
| --- | --- | --- |
| Diagnosis | n | % |
| F40-48 | 804852 | 78,34% |
| F30-F39 | 586637 | 57,10% |
| F10-F19 | 325099 | 31,64% |
| F60-69 | 184446 | 17,95% |
| F90-F98 | 175838 | 17,12% |
| F20-F29 | 156769 | 15,26% |
| F50-99 | 53142 | 5,17% |
| F80-89 | 32293 | 3,14% |
