# Supplementary material for: Mortality in people with mental disorders in Poland: A nationwide, register-based cohort study
Source: Eur Psychiatry. 2022 Nov 18;66(1):e2. doi: 10.1192/j.eurpsy.2022.2341 (PMC9879895; doi:10.1192/j.eurpsy.2022.2341)
Supplement: Supplementary file 1 [file S0924933822023410sup001.zip › S0924933822023410sup006.docx]

**Supplementary Table 2.** Sociodemographic characteristics of the study population

|  | |  | **Study population**  ***N* (%)** | **Deaths**  ***N* (%)** | |
| --- | --- | --- | --- | --- | --- |
| Sex | |  |  |  | |
|  | | Male | 1,811,336 (44.85%) | 31,394 (61.98%) | |
|  | | Female | 2,227,181 (55.15%) | 19,260 (38.02%) | |
| Age group [years] | |  |  |  | |
|  | | 15–24 | 40,1703 (9.95%) | 470 (0.93%) | |
|  | | 25–34 | 597,778 (14.80%) | 1614 (3.19%) | |
|  | | 35–44 | 823,412 (20.39%) | 4225 (8.34%) | |
|  | | 45–54 | 751,267 (18.60%) | 7312 (14.44%) | |
|  | | 55–64 | 809,569 (20.05%) | 14,700 (29.02%) | |
|  | | 65–74 | 500,061 (12.38%) | 14,083 (27.80%) | |
|  | | 75–84 | 154,727 (3.83%) | 8250 (16.29%) | |
| Diagnostic group  (ICD-10) | |  |  |  | |
|  | F10–19 | Mental and behaviour disorders due to psychoactive substance use | 709,707 (17.57%) | 20,188 (39.85%) | |
|  | F20–29 | Schizophrenia, schizotypal, delusional, and other non-mood psychotic disorders | 183,191 (4.54%) | 4490 (8.86%) | |
|  | F30–39 | Mood [affective] disorders | 427,655 (10.59%) | 5773 (11.40%) | |
|  | F40–48 | Anxiety, dissociative, stress-related, somatoform and other nonpsychotic mental disorders | 1,399,629 (34.66%) | 7904 (15.60%) | |
|  | F50-F59 | Behaviour syndromes associated with physiological disturbances and physical factors | 40,849 (1.01%) | 362 (0.71%) |  |
|  | F60-F69 | Disorders of adult personality and behaviour | 63,737 (1.58%) | 367 (0.72%) |  |
|  | F80-F89 | Pervasive and specific developmental disorders | 20,272 (0.50%) | 30 (0.06%) |  |
|  | F90-F99 | Behaviour and emotional disorders with onset usually occurring in childhood and adolescence | 166,130 (4.11%) | 753 (1.49%) |  |
|  |  | Individuals with a history of multiple diagnoses | 1,027,347 (25.44%) | 10,787 (21.30%) |  |
| Place of residence^a^ | |  |  |  |  |
|  | | Village | 1,331,713 (32.98%) | 9395 (18.55%) |  |
|  | | City with a population of less than or equal to 50,000 | 1,131,718 (28.02%) | 15,138 (29.89%) |  |
|  | | City with a population of more than 50,000 and less than or equal to 200,000 | 805,471 (19.94%) | 12,905 (25.48%) |  |
|  | | City with a population of more than 200,000 | 1,006,645 (24.93%) | 12,755 (25.18%) |  |
|  |  | Place of residence undefined | 342 (0.01%) | 461 (0.91%) |  |

*Note:* ^a^ – the sum of percentages exceeds 100 because more than one place of residence was shown for some patients
