# Supplementary material for: Mortality in people with mental disorders in Poland: A nationwide, register-based cohort study
Source: Eur Psychiatry. 2022 Nov 18;66(1):e2. doi: 10.1192/j.eurpsy.2022.2341 (PMC9879895; doi:10.1192/j.eurpsy.2022.2341)
Supplement: Supplementary file 1 [file S0924933822023410sup001.zip › S0924933822023410sup007.docx]

**Supplementary Table 3.** Distribution of deaths in general and study population according to age and sex

| **Age group** | **Number of deaths in psychiatric population** | **Number of deaths in general population** | **Proportion of deaths in psychiatric population** | **Proportion of deaths in general population** | **p–value** | **χ2** | **Power of test** |
| --- | --- | --- | --- | --- | --- | --- | --- |
| 0–14 | 34 | 2246 | 0.000608458 | 0.00536544 | 0 | 232.3 | 1 |
| 15–24 | 428 | 1895 | 0.007659407 | 0.004526941 | 0 | 986 | 0.9999993 |
| 25–34 | 1539 | 5035 | 0.027541652 | 0.012028046 | 0 | 867.2 | 1 |
| 35–44 | 4035 | 11,231 | 0.072209596 | 0.026829589 | 0 | 3258.9 | 1 |
| 45–54 | 7181 | 21,854 | 0.128509816 | 0.052206734 | 0 | 4994.9 | 1 |
| 55–64 | 14,304 | 60,107 | 0.255981675 | 0.143588825 | 0 | 4708.7 | 1 |
| 65–74 | 14,529 | 94,814 | 0.260008232 | 0.226499922 | 0 | 311.9 | 1 |
| 75–84 | 8270 | 102,07 | 0.147998354 | 0.243833686 | 0 | 2536.5 | 1 |
| 85+ | 5559 | 119,353 | 0.099482811 | 0.285120818 | 0 | 87 583 | 1 |

| **Age group** | **Sex** | **Number of deaths in psychiatric population** | **Number of deaths in general population** | **Proportion of deaths in psychiatric population** | **Proportion of deaths in general population** | **p–value** | **χ2** | **Power of test** |
| --- | --- | --- | --- | --- | --- | --- | --- | --- |
| 0–14 | W | 16 | 1034 | 0.000286111 | 0.002410109 | 0 | 105.5 | 1 |
| 0–14 | M | 18 | 1212 | 0.000122125 | 0.002895331 | 0 | 125.3 | 1 |
| 15–24 | W | 113 | 436 | 0.002022227 | 0.001041555 | 0 | 40.2 | 0.988918 |
| 15–24 | M | 315 | 1459 | 0.00563718 | 0.003485386 | 0 | 60.7 | 0.999682 |
| 25–34 | W | 285 | 992 | 0.00474239 | 0.002369776 | 0 | 104.l | 0.999999 |
| 25–34 | M | 1274 | 4043 | 0.022799263 | 0.00965827 | 0 | 767.l | 1 |
| 35–44 | W | 851 | 2693 | 0.015229335 | 0.006433272 | 0 | 511.1 | 1 |
| 35–44 | M | 3184 | 8538 | 0.056980261 | 0.020396316 | 0 | 2736.9 | 1 |
| 45–54 | W | 1823 | 5975 | 0.032624063 | 0.014273599 | 0 | 1025.8 | 1 |
| 45–54 | M | 5358 | 15,879 | 0.095885753 | 0.037933135 | 0 | 1871.2 | 1 |
| 55–64 | W | 4551 | 18,868 | 0.081443834 | 0.045073518 | 0 | 1389.l | 1 |
| 55–64 | M | 9753 | 41,239 | 0.174537841 | 0.098515307 | 0 | 2969.6 | 1 |
| 65–74 | W | 6101 | 35,960 | 0.10918234 | 0.085904373 | 0 | 130.4 | 1 |
| 65–74 | M | 8428 | 58,854 | 0.150825892 | 0.14059555 | 0 | 42.3 | 0.998058 |
| 75–84 | W | 5283 | 53,500 | 0.094543567 | 0.127805449 | 0 | 5 022 | 1 |
| 75–84 | M | 2987 | 48,570 | 0.053454786 | 0.116028237 | 0 | 1992.3 | 1 |
| 85+ | W | 4176 | 82,280 | 0.074732905 | 0.196557614 | 0 | 4909.2 | 1 |
| 85+ | M | 1383 | 37,073 | 0.024749906 | 0.088563204 | 0 | 2694.5 | 1 |

*Note:* The numerical data about the population aged 0-14 only appeared in the Supplementary Table 3 to show the distribution of the proportion of deaths in each age group. No analysis for this group has been performed.
