# Supplementary material for: Mortality in people with mental disorders in Poland: A nationwide, register-based cohort study
Source: Eur Psychiatry. 2022 Nov 18;66(1):e2. doi: 10.1192/j.eurpsy.2022.2341 (PMC9879895; doi:10.1192/j.eurpsy.2022.2341)
Supplement: Supplementary file 1 [file S0924933822023410sup001.zip › S0924933822023410sup008.docx]

**Supplementary Table 4.** Distribution of mortality in 2019 among people with a history of receiving healthcare services for mental and behavioural disorders in 2009–2018 who were alive on 1 January 2019, according to treatment settings, diagnoses of mental disorders, sex and age (numbers are expressed as a percentage of all deaths in the study population)

**Individuals with a history of multiple diagnoses**

|  | Outpatients - clinic/consultations | | | Outpatients - day care centre | | | Inpatients | | |
| --- | --- | --- | --- | --- | --- | --- | --- | --- | --- |
| **Female** | Deaths *N* | (%) | SMR (95%CI) | Deaths *N* | (%) | SMR | Deaths *N* | (%) | SMR |
| 15-24 | 20 | 0·04 | 2·29 (1·29-3·30) | 2 | 0·00 | 3·86 (-1·49-9·20) | 34 | 0·07 | 7·37 (4·89-9·84) |
| 25-34 | 26 | 0·05 | 1·25 (0·77-1·74) | 5 | 0·01 | 2·42 (0·30-4·54) | 60 | 0·12 | 7·39 (5·52-9·26) |
| 35-44 | 113 | 0·22 | 1·59 (1·29-1·88) | 9 | 0·02 | 1·32 (0·46-2·17) | 199 | 0·39 | 8·94 (7·70-10·19) |
| 45-54 | 280 | 0·55 | 1·41 (1·24-1·57) | 27 | 0·05 | 1·59 (0·99-2·19) | 310 | 0·61 | 4·75 (4·23-5·28) |
| 55-64 | 733 | 1·45 | 1·09 (1·01-1·17) | 49 | 0·10 | 1·02 (0·73-1·30) | 640 | 1·26 | 2·65 (2·45- 2·86) |
| 65-74 | 908 | 1·79 | 0·97 (0·90-1·03) | 28 | 0·05 | 0·84 (0·53-1·15) | 558 | 1·10 | 2·16 (1·98 - 2·34) |
| 75-84 | 740 | 1·46 | 0·83 (0·77-0·89) | 14 | 0·03 | 0·71(0·34-1·07) | 306 | 0·60 | 1·46 (1·29-1·62) |
| **Male** |  |  |  |  |  |  |  |  |  |
| 15-24 | 40 | 0·08 | 1·35 (0·93-1·77) | 2 | 0·00 | 0·89 (-0·34-2·13) | 93 | 0·18 | 6·56 (5·23-7·90) |
| 25-34 | 62 | 0·12 | 1·17 (0·88-1·46) | 6 | 0·01 | 1·08 (0·22-1·94) | 310 | 0·61 | 6·14(5·46-6·83) |
| 35-44 | 136 | 0·27 | 1·18 (0·98-1·37) | 5 | 0·01 | 0·42 (0·05-0·79) | 508 | 1·00 | 4·77 (4·36-5·19) |
| 45-54 | 301 | 0·59 | 1·24 (1·10-1·38) | 24 | 0·05 | 0·99 (0·59-1·38) | 669 | 1·32 | 3·20 (2·96-3·45) |
| 55-64 | 702 | 1·39 | 1·05 (0·97-1·13) | 47 | 0·09 | 0·93 (0·67-1·20) | 1023 | 2·02 | 2·09 (1·96-2·22) |
| 65-74 | 673 | 1·33 | 0·91 (0·84-0·97) | 34 | 0·07 | 0·89 (0·59-1·19) | 643 | 1·27 | 1·71 (1·58-1·85) |
| 75-84 | 278 | 0·55 | 0·81 (0·71-0·90) | 6 | 0·01 | 0·76 (0·15-1·37) | 164 | 0·32 | 1·52 (1·30-1·76) |
| SMR | 1·00 (0·98-1·03) | | | 0·96 (0·84-1·08) | | | 2·55 (2·48-2·62) | | |

**F10–19 Mental and behaviour disorders due to psychoactive substance use**

| Female | | | Outpatients - clinic/consultations | | | Outpatients - day care centre | | | Inpatients | | |
| --- | --- | --- | --- | --- | --- | --- | --- | --- | --- | --- | --- |
|  | Deaths *N* | | | (%) | SMR | Deaths *N* | (%) | SMR | Deaths *N* | (%) | SMR |
| 15-24 | | 6 | | 0·01 | 7·81 (1·56-14·06) | 1 | 0·00 | 43·34 (-41·61-128·29) | 3 | 0·01 | 7·94 (-1·04-16·93) |
| 25-34 | | 34 | | 0·07 | 11·06 (7·34-14·78) | 0 | 0·00 | 0.00 (0-0) | 55 | 0·11 | 28·07 (20·65-35·49) |
| 35-44 | | 106 | | 0·21 | 9·09 (7·36-10·82) | 16 | 0·03 | 12·44 (6·34-18·54) | 183 | 0·36 | 21·35 (18·26-24·45) |
| 45-54 | | 220 | | 0·43 | 6·41 (5·57-7·26) | 25 | 0·05 | 6·73 (4·09-9·37) | 303 | 0·60 | 11·18 (9·92-12·44) |
| 55-64 | | 449 | | 0·89 | 4·27 (3·87-4·66) | 42 | 0·08 | 4·39 (3·06-5·72) | 539 | 1·06 | 6·68 (6·11-7·24) |
| 65-74 | | 314 | | 0·62 | 2·53 (2·25-2·81) | 24 | 0·05 | 2·83 (1·7-3·97) | 339 | 0·67 | 3·96 (3·53-4·38) |
| 75-84 | | 119 | | 0·23 | 1·30 (1·06-1·53) | 3 | 0·01 | 1·56 (-0·20-3·32) | 95 | 0·19 | 2·35 (1·88-2·82) |
| Male | | |  |  |  |  |  |  |  |  |  |
| 15-24 | | 36 | | 0·07 | 4·54 (3·06-6·03) | 0 | 0·00 | 0·00 (0-0) | 41 | 0·08 | 8·28 (5·75-10·82) |
| 25-34 | | 221 | | 0·44 | 3·43 (2·98-3·88) | 14 | 0·03 | 3·84 (1·83-5·86) | 481 | 0·95 | 7·93 (7·22 - 8·64) |
| 35-44 | | 636 | | 1·26 | 3·36 (3·10-3·62) | 52 | 0·10 | 3·93 (2·86-5·00) | 1582 | 3·12 | 7·70 (7·32-8·08) |
| 45-54 | | 1122 | | 2·21 | 2·70 (2·54-2·86) | 92 | 0·18 | 2·74 (2·18-3·31) | 2429 | 4·79 | 5·13 (4·92-5·33) |
| 55-64 | | 2228 | | 4·40 | 2·20 (2·11-2·29) | 185 | 0·36 | 2·31 (1·97-2·64) | 3518 | 6·94 | 3·14 (3·03-3·24) |
| 65-74 | | 1627 | | 3·21 | 1·88 (1·79-1·97) | 109 | 0·21 | 1·62 (1·31-1·92) | 2208 | 4·36 | 2·51 (2·40-2·61) |
| 75-84 | | 346 | | 0·68 | 1·40 (1·25-1·55) | 18 | 0·04 | 1·33 (0·71-1·94) | 367 | 0·72 | 1·60 (1·43 - 1·76) |
| SMR | | | 2·35 (2·30-2·41) | | | 2·45 (2·25-2·65) | | | 3·77 (3·70-3·84) | | |

**F20–29 Schizophrenia, schizotypal, delusional, and other non-mood psychotic disorders**

|  | Outpatients - clinic/consultations | | | Outpatients - day care centre | | | Inpatients | | |
| --- | --- | --- | --- | --- | --- | --- | --- | --- | --- |
| **Female** | Deaths *N* | (%) | SMR | Deaths *N* | (%) | SMR | Deaths *N* | (%) | SMR |
| 15-24 | 0 | 0·00 | 0·00 (0 - 0) | 0 | 0·00 | 0·00 (0-0) | 1 | 0·00 | 4·84 (-4·64-14·32) |
| 25-34 | 1 | 0·00 | 1·35 (-1·29-3·99) | 0 | 0·00 | 0·00(0-0) | 7 | 0·01 | 4·48 (1·16-7·81) |
| 35-44 | 11 | 0·02 | 2·67 (1·09-4·25) | 0 | 0·00 | 0·00(0-0) | 36 | 0·07 | 4·81 (3·24-6·38) |
| 45-54 | 47 | 0·09 | 2·85 (2·04-3·67) | 1 | 0·00 | 1·06 (-1·02-3·14) | 84 | 0·17 | 3·81 (2·99-4·62) |
| 55-64 | 200 | 0·39 | 2·14 (1·84-2·44) | 7 | 0·01 | 2·08 (0·54-3·62) | 312 | 0·62 | 3·38 (3·01-3·76) |
| 65-74 | 434 | 0·86 | 2·19 (1·98-2·39) | 5 | 0·01 | 1·55 (0·19-2·91) | 471 | 0·93 | 3·19 (2·90-3·48) |
| 75-84 | 466 | 0·92 | 1·90 (1·73-2·07) | 3 | 0·01 | 1·34 (-0·18-2·86) | 324 | 0·64 | 2·18 (1·94-2·42) |
| **Male** |  |  |  |  |  |  |  |  |  |
| 15-24 | 3 | 0·01 | 7·08 (-0·93-15·10) | 0 | 0·00 | 0·00 (0-0) | 4 | 0·01 | 3·50 (0·07-6·93) |
| 25-34 | 10 | 0·02 | 2·12 (0·81-3·43) | 0 | 0·00 | 0·00 (0-0) | 47 | 0·09 | 3·77 (2·69-4·85) |
| 35-44 | 41 | 0·08 | 2·16 (1·50-2·82) | 3 | 0·01 | 2·08 (-0·27-4·44) | 84 | 0·17 | 2·42 (1·90-2·93) |
| 45-54 | 75 | 0·15 | 1·57 (1·21-1·92) | 2 | 0·00 | 0·81 (-0·31-1·93) | 150 | 0·30 | 2·54 (2·13-2·95) |
| 55-64 | 264 | 0·52 | 1·59 (1·39-1·78) | 7 | 0·01 | 1·21 (0·31-2·10) | 367 | 0·72 | 2·36 (2·12-2·60) |
| 65-74 | 352 | 0·69 | 1·45 (1·29-1·60) | 9 | 0·02 | 1·72 (0·60-2·84) | 338 | 0·67 | 2·18 (1·94-2·41) |
| 75-84 | 190 | 0·37 | 1·31 (1·12-1·49) | 3 | 0·01 | 2·15 (-0·28-4·58) | 131 | 0·26 | 1·95 (1·62-2·28) |
| SMR | 1·76 (1·69-1·84) | | | 1·49 (1·03-1·95) | | | 2·60 (2·50-2·71) | | |

**F30–39 Mood [affective] disorders**

|  | Outpatients - clinic/consultations | | | Outpatients - day care centre | | | Inpatients | | |
| --- | --- | --- | --- | --- | --- | --- | --- | --- | --- |
| **Female** | Deaths *N* | (%) | SMR | Deaths *N* | (%) | SMR | Deaths *N* | (%) | SMR |
| 15-24 | 4 | 0·01 | 2·14 (0·04-4·23) | 0 | 0·00 | 0·00 (0-0) | 1 | 0·00 | 2·58 (-2·48-7·65) |
| 25-34 | 16 | 0·03 | 1·95 (0·99-2·90) | 1 | 0·00 | 7·33 (-7·04-21·71) | 2 | 0·00 | 3·26 (-1·26-7·77) |
| 35-44 | 38 | 0·07 | 1·15 (0·78-1·51) | 3 | 0·01 | 4·93 (-0·65-10·51) | 8 | 0·02 | 3·97 (1·22-6·72) |
| 45-54 | 126 | 0·25 | 1·16 (0·95-1·36) | 2 | 0·00 | 0·77 (-0·30-1·83) | 39 | 0·08 | 4·19 (2·87-5·50) |
| 55-64 | 508 | 1·00 | 1·02 (0·93-1·11) | 12 | 0·02 | 0·94 (0·41-1·48) | 123 | 0·24 | 1·82 (1·50-2·14) |
| 65-74 | 955 | 1·88 | 1·05 (0·98-1·11) | 10 | 0·02 | 0·80 (0·30-1·29) | 249 | 0·49 | 2·07 (1·81-2·32) |
| 75-84 | 1165 | 2·30 | 0·96 (0·90-1·01) | 13 | 0·03 | 0·75 (0·34-1·16) | 369 | 0·73 | 1·76 (1·58-1·94) |
| **Male** |  |  |  |  |  |  |  |  |  |
| 15-24 | 12 | 0·02 | 4·25 (1·84-6·65) | 1 | 0·00 | 18·93 (-18·17-56·02) | 1 | 0·00 | 1·95 (-1·87-5·76) |
| 25-34 | 20 | 0·04 | 1·24 (0·70-1·78) | 0 | 0·00 | 0·00 (0-0) | 7 | 0·01 | 2·99 (0·77-5·20) |
| 35-44 | 43 | 0·08 | 1·07 (0·75-1·39) | 0 | 0·00 | 0·00 (0-0) | 12 | 0·02 | 2·37 (1·03-3·71) |
| 45-54 | 98 | 0·19 | 0·94 (0·75-1·13) | 0 | 0·00 | 0·00 (0-0) | 33 | 0·06 | 1·90 (1·25-2·55) |
| 55-64 | 397 | 0·78 | 0·96 (0·86-1·05) | 9 | 0·02 | 0·76 (0·26-1·26) | 125 | 0·25 | 1·38 (1·13-1·62) |
| 65-74 | 623 | 1·23 | 0·90 (0·83-0·97) | 4 | 0·01 | 0·26 (0·00-0·52) | 185 | 0·36 | 1·25 (1·07-1·43) |
| 75-84 | 408 | 0·80 | 0·89 (0·80-0·97) | 7 | 0·01 | 1·21 (0·31-2·10) | 144 | 0·28 | 1·49 (1·25-1·74) |
| SMR | 0·98 (0·95-1·01) | | | 0·75 (0·57-0·94) | | | 1·68 (1·59-1·78) | | |

**F40–48 Anxiety, dissociative, stress-related, somatoform and other nonpsychotic mental disorders**

|  | Outpatients - clinic/consultations | | | Outpatients - day care centre | | | Inpatients | | |
| --- | --- | --- | --- | --- | --- | --- | --- | --- | --- |
| **Female** | Deaths *N* | (%) | SMR | Deaths *N* | (%) | SMR | Deaths *N* | (%) | SMR |
| 15-24 | 14 | 0·02 | 1·17 (0·56-1·79) | 0 | 0·00 | 0·00 (0-0) | 7 | 0·01 | 7·54 (1·95-13·12) |
| 25-34 | 48 | 0·09 | 0·88 (0·63-1·13) | 0 | 0·00 | 0·00 (0-0) | 7 | 0·01 | 3·44 (0·89-5·98) |
| 35-44 | 149 | 0·29 | 0·81 (0·68-0·94) | 3 | 0·01 | 0·66 (-0·09-1·40) | 15 | 0·03 | 3·19 (1·58-4·80) |
| 45-54 | 362 | 0·71 | 0·83 (0·75-0·92) | 9 | 0·02 | 0·92 (0·32-1·51) | 18 | 0·04 | 1·27 (0·68-1·86) |
| 55-64 | 981 | 1·94 | 0·81 (0·76-0·86) | 10 | 0·02 | 0·45 (0·17-0·72) | 77 | 0·15 | 1·57 (1·22-1·93) |
| 65-74 | 1402 | 2·77 | 0·80 (0·76-0·84) | 13 | 0·03 | 0·81 (0·37-1·25) | 115 | 0·23 | 1·82 (1·49-2·15) |
| 75-84 | 1263 | 2·49 | 0·76 (0·72-0·80) | 9 | 0·02 | 0·73 (0·25-1·20) | 162 | 0·32 | 2·01 (1·70-2·32) |
| **Male** |  |  |  |  |  |  |  |  |  |
| 15-24 | 22 | 0·04 | 0·95 (0·55-1·34) | 0 | 0·00 | 0·00(0-0) | 8 | 0·02 | 4·64 (1·42-7·86) |
| 25-34 | 76 | 0·15 | 0·77 (0·60-0·94) | 0 | 0·00 | 0·00(0-0) | 26 | 0·05 | 3·07 (1·89-4·25) |
| 35-44 | 139 | 0·27 | 0·57 (0·48-0·67) | 4 | 0·01 | 0·53 (0·01-1·04) | 29 | 0·06 | 1·39 (0·88-1·90) |
| 45-54 | 309 | 0·61 | 0·62 (0·55-0·69) | 6 | 0·01 | 0·25 (0·05-0·44) | 57 | 0·11 | 0·76 (0·57-0·96) |
| 55-64 | 771 | 1·52 | 0·66 (0·61-0·70) | 13 | 0·03 | 0·46 (0·21-0·70) | 115 | 0·23 | 0·89 (0·73-1·05) |
| 65-74 | 937 | 1·85 | 0.72 (0.67-0.77) | 7 | 0·01 | 0·48 (0·12-0·84) | 113 | 0·22 | 1·40 (1·14-1·66) |
| 75-84 | 529 | 1·04 | 0·75 (0·69-0·81) | 5 | 0·01 | 1·02 (0·13-1·92) | 74 | 0·15 | 1·70 (1·31-2·08) |
| SMR | 0·53 (0·41-0·65) | | | 0·75 (0·73-0·77) | | | 1·43 (1·34-1·53) | | |

**F50-F59 Behaviour syndromes associated with physiological disturbances and physical factors**

|  | Outpatients - clinic/consultations | | | Outpatients - day care centre | | | Inpatients | | | |
| --- | --- | --- | --- | --- | --- | --- | --- | --- | --- | --- |
| **Female** | Deaths *N* | (%) | SMR | Deaths *N* | (%) | SMR | Deaths *N* | (%) | SMR |  |
| 15-24 | 1 | 0·00 | 0·85 (-0·81-2·51) | 0 | 0·00 | 0·00 (0-0) | 3 | 0·01 | 10·13 (-1·33-21·60) |  |
| 25-34 | 2 | 0·00 | 0·97 (-0·37-2·32) | 0 | 0·00 | 0·00 (0-0) | 1 | 0·00 | 4·19 (-4·02-12·41) |  |
| 35-44 | 1 | 0·00 | 0·40 (-0·39-1·19) | 0 | 0·00 | 0·00 (0-0) | 0 | 0·00 | 0·00 (0-0) |  |
| 45-54 | 4 | 0·01 | 0·91 (0·02-1·79) | 0 | 0·00 | 0·00 (0-0) | 0 | 0·00 | 0·00 (0-0) |  |
| 55-64 | 20 | 0·04 | 1·19 (0·67-1·72) | 0 | 0·00 | 0·00 (0-0) | 1 | 0·00 | 5·95 (-5·71-17·62) |  |
| 65-74 | 44 | 0·09 | 0·91 (0·64-1·18) | 0 | 0·00 | 0·00 (0-0) | 1 | 0·00 | 2·06 (-1·98-6·10) |  |
| 75-84 | 69 | 0·14 | 0·73 (0·56-0·90) | 1 | 0·00 | 1·76 (-1·69-5·20) | 5 | 0·01 | 3·46 (0·43-6·49) |  |
| **Male** |  |  |  |  |  |  |  |  |  |  |
| 15-24 | 3 | 0·01 | 2·20 (-0·29-4·69) | 0 | 0·00 | 0·00 (0-0) | 0 | 0·00 | 0·00 (0-0) |  |
| 25-34 | 2 | 0·00 | 0·62 (-0·24-1·48) | 0 | 0·00 | 0·00 (0-0) | 0 | 0·00 | 0·00 (0-0) |  |
| 35-44 | 3 | 0·01 | 0·49 (-0·06-1·04) | 0 | 0·00 | 0·00 (0-0) | 0 | 0·00 | 0·00 (0-0) |  |
| 45-54 | 13 | 0·03 | 1·06 (0·48-1·63) | 0 | 0·00 | 0·00 (0-0) | 0 | 0·00 | 0·00 (0-0) |  |
| 55-64 | 28 | 0·05 | 0·73 (0·46-1·00) | 0 | 0·00 | 0·00 (0-0) | 3 | 0·01 | 5·12 (-0·67-10·92) |  |
| 65-74 | 61 | 0·12 | 0·66 (0·50-0·83) | 0 | 0·00 | 0·00 (0-0) | 8 | 0·02 | 4·82 (1·48-8·16) |  |
| 75-84 | 82 | 0·16 | 0·83 (0·65-1·01) | 1 | 0·00 | 2·39 (-2·29-7·07) | 5 | 0·01 | 2·47 (0·30-4·64) |  |
| SMR | 0·79 (0·70-0·87) | | | 0·98 (-0·38-2·34) | | | 3·60 (2·24-4·96) | | | |

**F60-F69 Disorders of adult personality and behaviour**

|  | Outpatients - clinic/consultations | | | | Outpatients - day care centre | | | Inpatients | | |
| --- | --- | --- | --- | --- | --- | --- | --- | --- | --- | --- |
| **Female** | | Deaths *N* | (%) | SMR | Deaths *N* | (%) | SMR | Deaths *N* | (%) | SMR |
| 15-24 | | 1 | 0·00 | 1·44 (-1·39-4·28) | 1 | 0·00 | 67·04 (-64·36-198·44) | 1 | 0·00 | 9·86 (-9·47-29·20) |
| 25-34 | | 2 | 0·00 | 0·69 (-0·27-1·65) | 0 | 0·00 | 0·00 (0-0) | 0 | 0·00 | 0·00 (0 - 0) |
| 35-44 | | 5 | 0·01 | 1·12 (0·14-2·10) | 1 | 0·00 | 3·65 (-3·51-10·81) | 1 | 0·00 | 2·73 (-2·62-8·07) |
| 45-54 | | 5 | 0·01 | 0·86 (0·11-1·62) | 0 | 0·00 | 0·00 (0-0) | 1 | 0·00 | 1·80 (-1·73-5·34) |
| 55-64 | | 20 | 0·04 | 1·61 (0·90-2·32) | 0 | 0·00 | 0·00 (0-0) | 2 | 0·00 | 1·93 (-0·74-4·61 |
| 65-74 | | 20 | 0·04 | 1·05 (0·59-1·51) | 2 | 0·00 | 6·95 (-2·68-16·57) | 6 | 0·01 | 3·96 (0·79-7·13) |
| 75-84 | | 28 | 0·05 | 1·24 (0·78-1·70) | 1 | 0·00 | 1·63 (-1·57-4·83) | 8 | 0·02 | 2·81 (0·86-4·76) |
| **Male** | |  |  |  |  |  |  |  |  |  |
| 15-24 | | 8 | 0·02 | 3·15 (0·97-5·33) | 0 | 0·00 | 0·00 (0-0) | 2 | 0·00 | 6·67 (-2·57-15·91) |
| 25-34 | | 22 | 0·04 | 1·30 (0·76-1·85) | 0 | 0·00 | 0·00 (0 - 0) | 13 | 0·03 | 4·55 (2·08-7·03) |
| 35-44 | | 20 | 0·04 | 0·81 (0·45-1·16) | 1 | 0·00 | 1·20 (-1·15-3·55) | 10 | 0·02 | 2·68 (1·02-4·34) |
| 45-54 | | 25 | 0·05 | 0·93 (0·56-1·29) | 3 | 0·01 | 3.70 (-0·49-7·89) | 6 | 0·01 | 1·58 (0·32-2·85) |
| 55-64 | | 47 | 0·09 | 0·99 (0·71-1·28) | 1 | 0·00 | 1·00 (-0·96-2·95) | 12 | 0·02 | 1·85 (0·80-2·90) |
| 65-74 | | 46 | 0·09 | 0·99 (0·70-1·27) | 0 | 0·00 | 0·00 (0-0) | 15 | 0·03 | 2·30 (1·14-3·47) |
| 75-84 | | 24 | 0·05 | 0·98 (0·59-1·38) | 0 | 0·00 | 0·00 (0-0) | 7 | 0·01 | 2·71 (0·70-4·72) |
| SMR | 1·06 (0·93-1·19) | | | | 1·64 (0·62-2·65) | | | 2·55 (2·00-3·09) | | |

**F80-F89 Pervasive and specific developmental disorders**

|  | Outpatients - clinic/consultations | | | Outpatients - day care centre | | | Inpatients | | |
| --- | --- | --- | --- | --- | --- | --- | --- | --- | --- |
| **Female** | Deaths *N* | (%) | SMR | Deaths *N* | (%) | SMR | Deaths *N* | (%) | SMR |
| 15-24 | 3 | 0·01 | 3·39 (-0·45-7·23) | 1 | 0 | 23·97 (-23·01-70·95) | 0 | 0·00 | 0·00 (0-0) |
| 25-34 | 2 | 0 | 8·46 (-3·27-20·19) | 0 | 0 | 0·00 (0-0) | 0 | 0·00 | 0·00 (0-0) |
| 35-44 | 0 | 0 | 0·00 (0-0) | 0 | 0 | 0·00 (0-0) | 0 | 0·00 | 0·00 (0-0) |
| 45-54 | 0 | 0 | 0·00(0-0) | 0 | 0 | 0·00 (0-0) | 1 | 0·00 | 81·57 (-78·30- 241·44) |
| 55-64 | 0 | 0 | 0·00(0-0) | 0 | 0 | - | 0 | 0·00 | - |
| 65-74 | 0 | 0 | 0·00(0-0) | 0 | 0 | - | 0 | 0·00 | 0·00 (0-0) |
| 75-84 | 1 | 0 | 3·26 (-3·13-9·66) | 0 | 0 | - | 0 | 0·00 | 0·00 (0-0) |
| **Male** |  |  |  |  |  |  |  |  |  |
| 15-24 | 10 | 0·02 | 1·15 (0·44-1·86) | 0 | 0·00 | 0·00 (0-0) | 1 | 0·00 | 2·37 (-2·28-7·03) |
| 25-34 | 3 | 0·01 | 1·27 (-0·17-2·71) | 1 | 0·00 | 8·42 (-8·08-24·92) | 1 | 0·00 | 4·34 (-4·17-12·86) |
| 35-44 | 1 | 0·00 | 1·52 (-1·46-4·51) | 0 | 0·00 | 0·00 (0-0) | 0 | 0·00 | 0·00 (0-0) |
| 45-54 | 1 | 0·00 | 2·01 (-1·93-5·96) | 1 | 0·00 | 38·25 (-36·72-113·22) | 0 | 0·00 | 0·00 (0-0) |
| 55-64 | 1 | 0·00 | 1·13 (-1·08-3·34) | 0 | 0·00 | - | 0 | 0·00 | - |
| 65-74 | 1 | 0·00 | 1·74 (-1·67-5·15) | 0 | 0·00 | - | 0 | 0·00 | 0·00 (0-0) |
| 75-84 | 1 | 0·00 | 2·87 (-2·75-8·48) | 0 | 0·00 | - | 0 | 0·00 | 0·00 (0-0) |
| SMR | 1·48 (0·89-2·08) | | | - | | | - | | |

**F90-F99 Behaviour and emotional disorders with onset usually occurring in childhood and adolescence**

|  | Outpatients - clinic/consultations | | | Outpatients - day care centre | | | Inpatients | | | |
| --- | --- | --- | --- | --- | --- | --- | --- | --- | --- | --- |
| **Female** | Deaths *N* | (%) | SMR | Deaths *N* | (%) | SMR | Deaths *N* | (%) | SMR |  |
| 15-24 | 10 | 0·02 | 1·13 (0·43-1·84) | 0 | 0·00 | 0·00 (0-0) | 4 | 0·01 | 3·78 (0·08-7·49) | |
| 25-34 | 4 | 0·01 | 1·38 (0·028-2·74) | 0 | 0·00 | 0·00 (0-0) | 2 | 0·00 | 9.06 (-3·50-21·62) | |
| 35-44 | 8 | 0·02 | 2·25 (0·69-3·82) | 0 | 0·00 | 0·00 (0-0) | 0 | 0·00 | 0·00 (0-0) | |
| 45-54 | 11 | 0·02 | 1·45 (0·59-2·31) | 0 | 0·00 | 0·00 (0-0) | 0 | 0·00 | 0·00 (0-0) | |
| 55-64 | 37 | 0·07 | 1·59 (1·08-2·11) | 0 | 0·00 | 0·00 (0-0) | 7 | 0·01 | 11·49 (2·98-20·01) | |
| 65-74 | 64 | 0·13 | 1·19 (0·90-1·48) | 0 | 0·00 | 0·00 (0-0) | 14 | 0·03 | 6·04 (2·87-9·20) | |
| 75-84 | 148 | 0·29 | 1.29 (1·08-1·5) | 2 | 0·00 | 3·51 (-1·36-8·38) | 28 | 0·05 | 3·87 (2·44 - 5·31) | |
| **Male** |  |  |  |  |  |  |  |  |  | |
| 15-24 | 48 | 0·09 | 0·99 (0·71-1·27) | 0 | 0·00 | 0·00(0-0) | 17 | 0·03 | 3·19 (1·67-4·70) | |
| 25-34 | 14 | 0·03 | 0·95 (0·45-1·45) | 0 | 0·00 | 0·00(0-0) | 3 | 0·01 | 2·07 (-0·27- 4·41) | |
| 35-44 | 10 | 0·02 | 1·21 (0·46-1·97) | 0 | 0·00 | 0·00(0-0) | 1 | 0·00 | 3·28 (-3·15-9·71) | |
| 45-54 | 20 | 0·04 | 1·38 (0·78-1·99) | 1 | 0·00 | 30·60 (-29·38-90·58) | 0 | 0·00 | 0·00 (0 - 0) | |
| 55-64 | 61 | 0·12 | 1·46 (1·09-1·82) | 1 | 0·00 | 3·51 (-3·37-10·40) | 6 | 0·01 | 2·62 (0·52-4·71) | |
| 65-74 | 101 | 0·20 | 1·29 (1·04-1·54) | 0 | 0·00 | 0·00(0-0) | 13 | 0·03 | 2·75 (1·26-4·25) | |
| 75-84 | 99 | 0·19 | 1·17 (0·94-1·40) | 0 | 0·00 | 0·00(0-0) | 19 | 0·04 | 3·63 (2·00-5·26) | |
| SMR | 1·26 (1·16-1·35) | | | 1·64 (0·62-2·65) | | | 2·55 (2·00-3·09) | | | |
