# Supplementary material for: Mortality in people with mental disorders in Poland: A nationwide, register-based cohort study
Source: Eur Psychiatry. 2022 Nov 18;66(1):e2. doi: 10.1192/j.eurpsy.2022.2341 (PMC9879895; doi:10.1192/j.eurpsy.2022.2341)
Supplement: Supplementary file 1 [file S0924933822023410sup001.zip › S0924933822023410sup009.docx]

**Supplementary Table 5**

*Differences in SMR/MRR values between our study and other studies*

| **SMR value in our study (95% CI)** | **SMR/MRR values in other studies (95% CI)** |
| --- | --- |
| **Any diagnosis - inpatients only** | |
| 2.9 (2.86-2.94) | Krupchanka et al. [11]: 2.2 (2.2-2.3)  Park et al. [34]: 2.14 (2.00-2.28) |
| **Any diagnosis - inpatients and outpatients** | |
| 1.54 (1.53-1.55) | Plana-Ripoll et al. [6]: 2.53 (2.52-2.54) Berardi et al. [21]: 1.99 (1.96-2.03)  Starace et al. [22]: 1.8 (1.7-1.9) Termorshuizen et al. [23]: 2.99 (2.63-3.41)  Park et al. [34]: 1.62 (1.57-1.68) |
| **Any diagnosis - outpatients only** | |
| Outpatients - clinic/consultations: 1.12 (1.10-1.13) Outpatients - day care centre: 1.34 (1.26-1.42) | Park et al. [34]: 1.48 (1.43-1.55) |
