# Supplementary figures and images for: Mortality in people with mental disorders in Poland: A nationwide, register-based cohort study
Source: Eur Psychiatry. 2022 Nov 18;66(1):e2. doi: 10.1192/j.eurpsy.2022.2341 (PMC9879895; doi:10.1192/j.eurpsy.2022.2341)

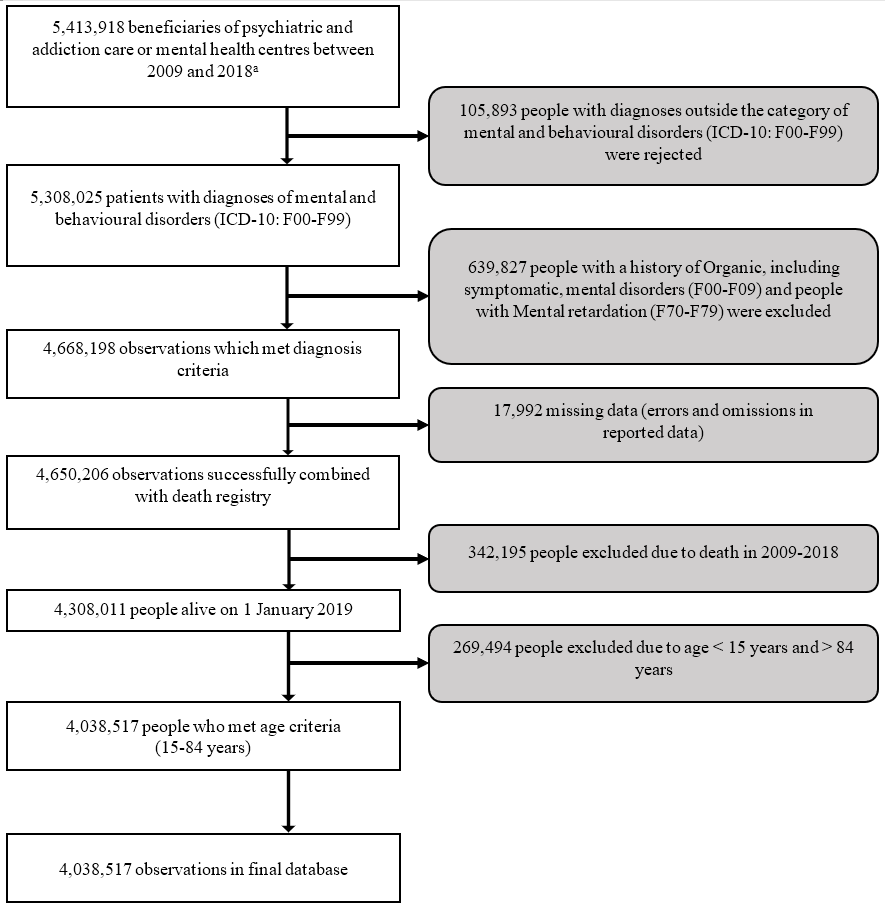

Supplement: Supplementary file 1 [file S0924933822023410sup001.zip › S0924933822023410sup001.tif]

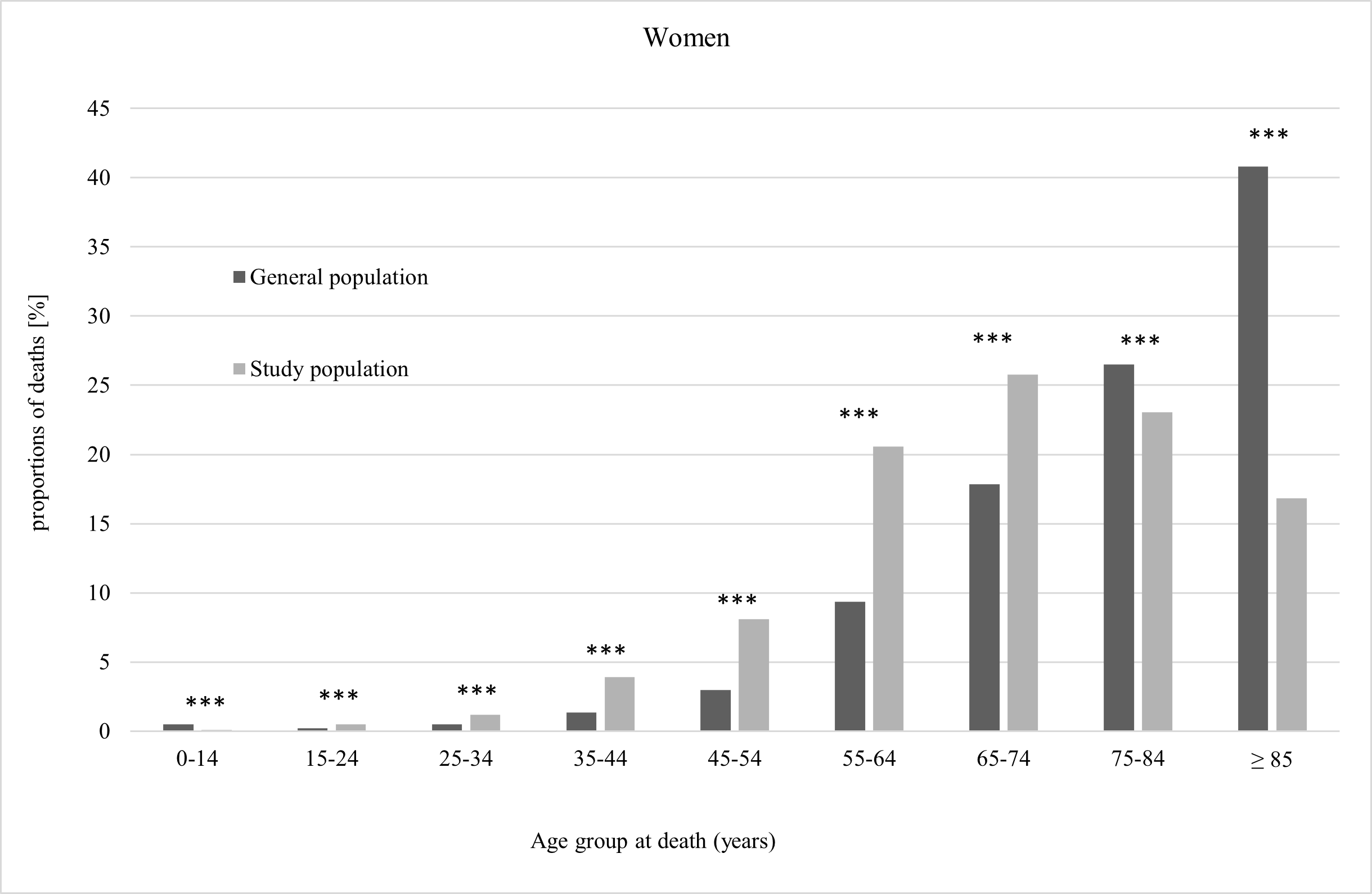

Supplement: Supplementary file 1 [file S0924933822023410sup001.zip › S0924933822023410sup002.tif]

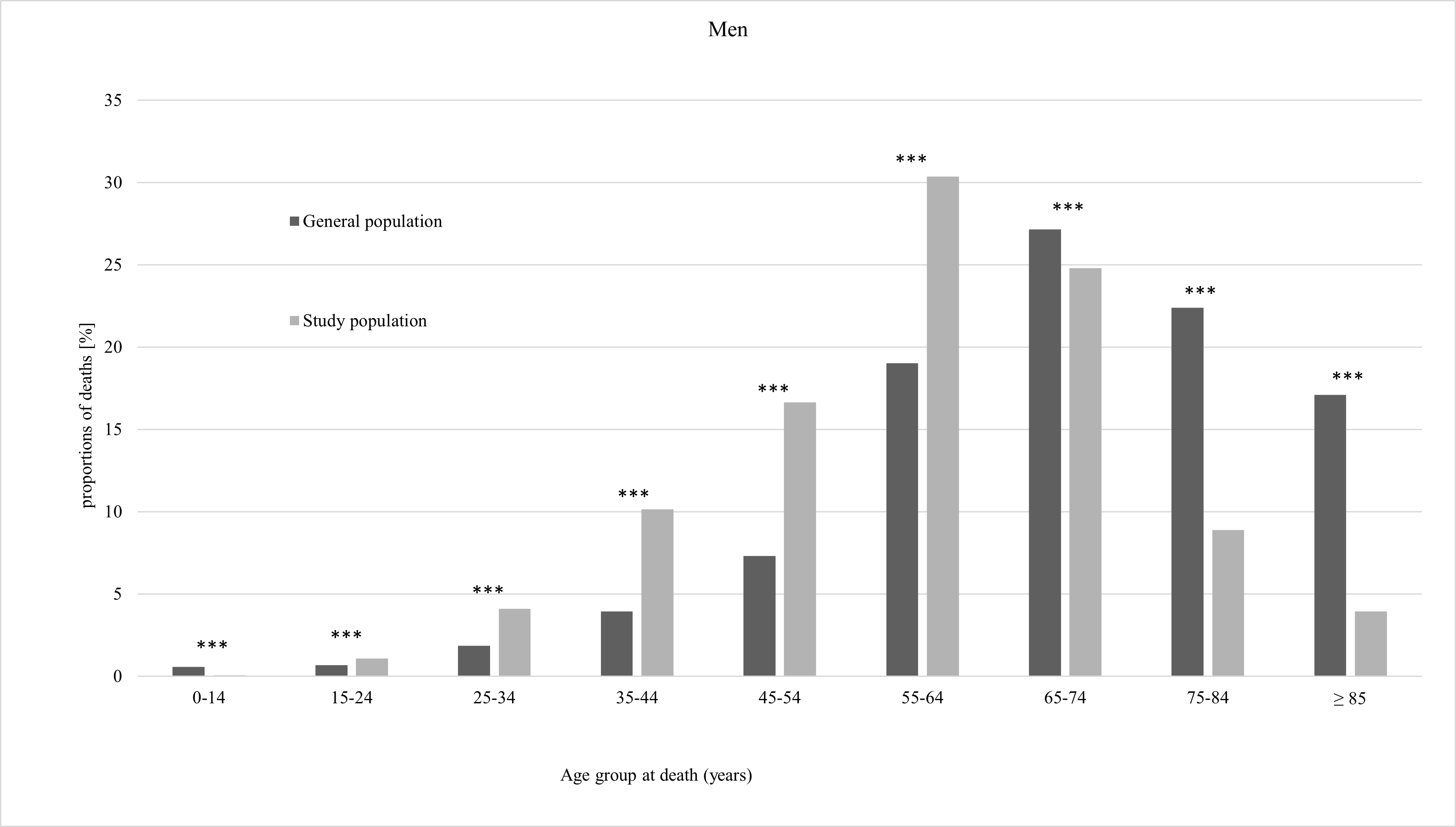

Supplement: Supplementary file 1 [file S0924933822023410sup001.zip › S0924933822023410sup003.tif]
